# Escaping the symmetry trap in helical reconstruction

**DOI:** 10.1039/d2fd00051b

**Published:** 2022-08-05

**Authors:** Lavinia Gambelli, Michail N. Isupov, Bertram Daum

**Affiliations:** a College of Engineering, Mathematics and Physical Sciences, University of Exeter Exeter EX4 4QF UK; b Living Systems Institute, University of Exeter Exeter EX4 4QD UK b.daum2@exeter.ac.uk; c Henry Wellcome Building for Biocatalysis, Biosciences, College of Life and Environmental Sciences, University of Exeter Exeter EX4 4QD UK; d College of Life and Environmental Sciences, University of Exeter Exeter EX4 4QD UK

## Abstract

Helical reconstruction is the method of choice for obtaining 3D structures of filaments from electron cryo-microscopy (cryoEM) projections. This approach relies on applying helical symmetry parameters deduced from Fourier–Bessel or real space analysis, such as sub-tomogram averaging. While helical reconstruction continues to provide invaluable structural insights into filaments, its inherent dependence on imposing a pre-defined helical symmetry can also introduce bias. The applied helical symmetry produces structures that are infinitely straight along the filament’s axis and can average out biologically important heterogeneities. Here, we describe a simple workflow aimed at overcoming these drawbacks in order to provide truer representations of filamentous structures.

## Abstract 2

In this study, we reflect on widely-used helical processing routines in cryoEM and suggest a new workflow capable of uncovering heterogeneities and complex symmetries that could otherwise be overlooked.

## Graphical abstract

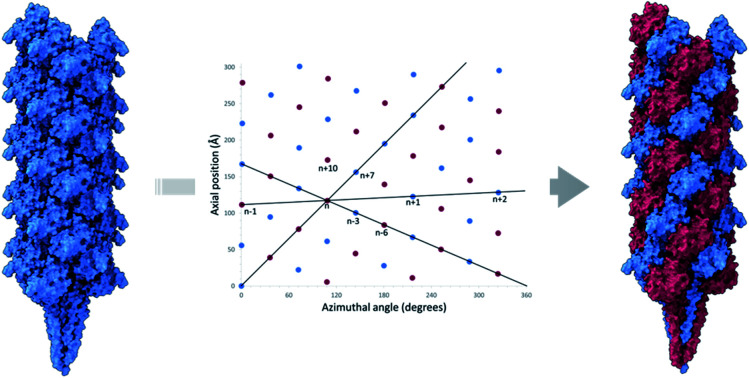

## Introduction

Filamentous protein assemblies are found everywhere in biology and play key roles in many aspects of cellular life. For example, actin^1^ and microtubule^2^ filaments form the cytoskeleton in most eukaryotic cells, flagella^3^ and archaella^4^ propel bacteria and archaea through liquid, and helical capsids enclose filamentous viruses.^5^

Electron cryo-microscopy (cryoEM) has enabled the structural determination of a plethora of protein filaments through an image processing approach called helical reconstruction. For this, a sample of purified filaments is vitrified on electron microscopy grids and usually hundreds to thousands of micrographs are then recorded in an electron microscope. Since most biological filaments have intrinsic helical symmetry, each subunit within a filament adopts a different but defined orientation with respect to the image plane. This means that when the orientation and position of each filament subunit with respect to the filament’s axis is established, it becomes possible to calculate a 3-dimensional reconstruction.^6^

Classically, this approach involved the use of Fourier–Bessel analysis, whereby the diffraction pattern of a filament calculated by a Fourier transform of the image is used to determine the screw operations, or helical parameters. These helical parameters define the relative displacement of the symmetry-related subunits within the helix and are known as helical rise (the translational offset between adjacent asymmetrical units), twist (the angular offset between adjacent asymmetrical units), pitch (the length of a complete helix turn) and number of subunits per turn (NUT). If either rise and twist or pitch and NUT can be determined, the three-dimensional structure of the filament can be reconstructed in a process known as Fourier–Bessel inversion.^6,7^ However, the classical Fourier–Bessel method relies on high-quality diffraction patterns that are only obtained from highly ordered and relatively straight filaments – features that rarely apply to often undulating biological specimens. Moreover, the determination of the screw operations from diffraction patterns is sometimes precluded by the overlap of Bessel helical layer lines.^8,9^

More recently, single particle approaches have been developed, which do not depend on high quality diffraction of the raw data. This strategy was first implemented with IHRSR,^9^ later in SPRING^10^ and more recently in the Relion^11^ and cryoSPARC^12^ software packages. In a nutshell, the filaments are first divided up into segments, which are then initially treated as single particles, meaning that they are aligned with a reference and averaged to yield a projection of the reference with greatly enhanced signal to noise ratio. As in the single particle approach, the data can be classified to 2D classes to account for heterogeneity in the sample.^13^ To obtain the screw operators that are important for the 3D reconstruction, the 2D class averages are analysed both in real and Fourier space. For filaments of low order, rise and pitch values can sometimes be estimated from the projection. However, analyzing the layer lines of the diffraction pattern in Fourier space usually gives more precise clues and is the only source for estimating the twist value from the projection data.^9^ By applying the screw operators, the data are then iteratively aligned with a reference and back-projected into a 3D map. In this process, the helical parameters can be interactively refined until the reconstruction reaches its final resolution (does not improve with additional iterations).^9^

Unfortunately, layer line profiles can often be inconclusive (*e.g.* through Bessel overlap or appear to suggest more than one solution to the helical parameters).^8,9^ In those cases, the layer line profiles are used to narrow down sets of helical parameters that then need to be tested. This is aided by helical symmetry search routines implemented in software such as SPRING^11^ and cryoSPARC.^12^ During this process, all sets of helical parameters that do not produce a biologically meaningful 3D reconstruction are discarded and the set that does is considered as correct. For example, at a Gold-Standard resolution under ∼4.6 Å, secondary structure features (α-helices, β-strands and loops) should be resolved and α-helices should be right handed. These maps are then taken forward to build an atomic model and further interpretation.

Using the structure of an archaellum as an example, we highlight how this approach can be a trap, as more complex symmetries can be disguised by seemingly correct helical parameters. We further suggest a way out of this trap, whichcould be useful as a default step for any helical reconstruction project.

## The symmetry trap – a showcase

**Fig. 1:**
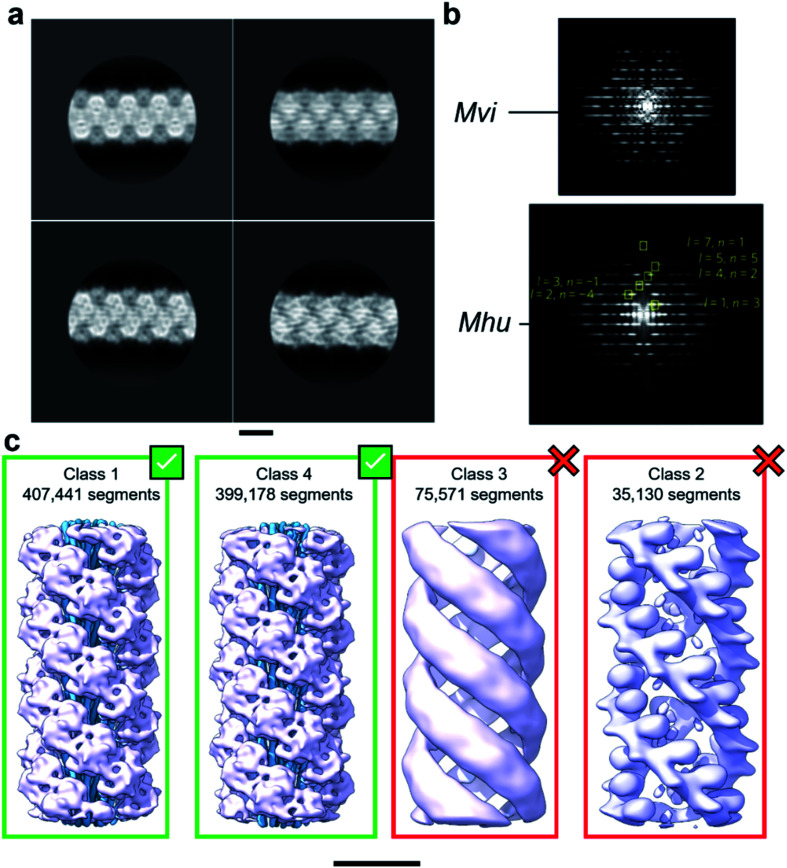
Helical reconstruction of archaella from *M. villosus*. (a) Examples of 2D classification of the *M. villosus* archaellum obtained in Relion 3.1. (b) Layer line profiles of the *M. villosus* (*Mvi*, top) and *M. hungatei* (*Mhu*, bottom [re-used from ref. 17 with permission]) filaments. (c) Examples of 3D classifications obtained in Relion 3.1. Classes 1 and 4, which show typical features of archaella (central α-helix bundles (blue) and peripheral globular domains (mauve)), were selected for further processing. Scale bar, 50 Å.

**Fig. 2:**
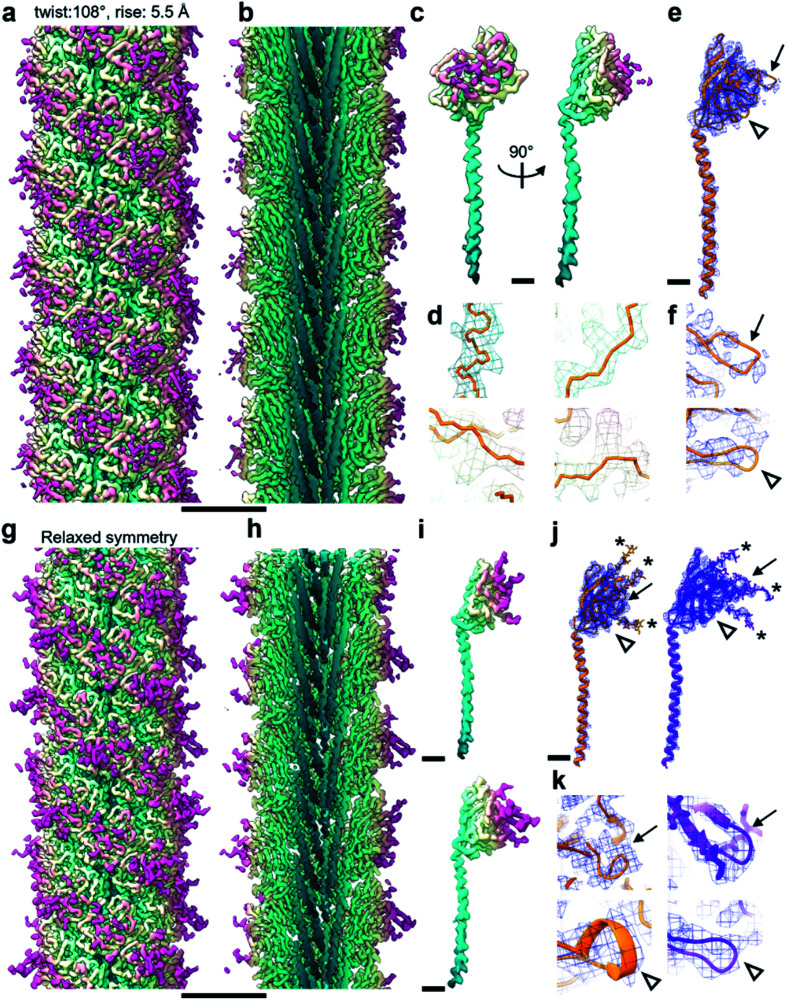
Relaxing the symmetry reveals an improved map. (a and b) CryoEM map viewed from outside (a) and its cross-section (b) of the *M. villosus* archaellum filament solved imposing a helical symmetry with 108° twist and 5.5 Å rise. The map is coloured in petrol blue–light yellow–magenta from the core to the periphery of the filament. (c) CryoEM density forming the asymmetric unit of the filament in (a) and (b). (d) Close-ups of the cryoEM map in (c) showing large side chains densities in mesh and backbone tracing in orange. (e) CryoEM map from (c) as blue mesh with atomic model of ArlB2 as orange ribbon. The two arrows highlight areas in which the cryoEM map is fragmented. (f) Close-ups of the fragmented cryoEM areas. (g and h) CryoEM map (g) and cross-section (h) of the filament after symmetry relaxation. The colour scheme is the same as in (a). (i) CryoEM density of the two subunits forming the filament in (g). (j) CryoEM maps of the two subunits in (i) in blue mesh with atomic models in ribbon representation. ArlB1, orange; ArlB2, purple. (k) Close-ups of the areas in (j) in which the cryoEM map supports the atomic models of ArlB1 and ArlB2. The arrows in (j) and (k) point at the same areas as in (e) and (f). Comparison between (e), (f), (j) and (k) shows how the map improved through symmetry relaxation and contains additional information, such as glycan densities (* in (j) and (k)). Scale bar in (a, b, g, h), 50 Å; in (c, e, i, j), 10 Å.

With the aim to investigate the structure of the archaellum from the archaeal species *Methanocaldococcus villosus*, we recorded 2759 movies and extracted 929 165 segments in Relion 3.1.^14^ 2D classification provided classes with the typical appearance for archaella, hallmarked by a bundle of α-helices in the core of the filament (Fig. 1a). Fourier transforms of the 2D classes yielded layer line profiles seemingly consistent with helical parameters that appear to be conserved for archaella across species – with an apparent rise of ∼5.5 Å and a twist of ∼108° (Fig. 1b;^17^). Applying these parameters, we performed 3D classification (Fig. 1c), chose the best resolved class containing 399 178 segments and subjected those particles to 3D refinement, whereby the helical parameters were autorefined to 5.57 Å rise and 108° twist.^14^ After CTF refinement and Bayesian polishing, the shiny particles were used for a last round of refinement, resulting in a final resolution of 3.29 Å (Fig. 2a and b). The refined map had the typical structure of an archaellum, consisting of lollipop-shaped subunits with an α-helical “tail” and a β-strand rich globular “head” domain (Fig. 2c). As to be expected at this resolution, α-helices and β-strands were well resolved and large side chains were visible (Fig. 2c and d). Based on this map, we built an initial atomic model (Fig. 2e).^14^

Archaellum operons usually encode one to seven archaellum subunits called archaellins^15,16^ and based on previously published structures, it is believed that only one of these archaellins assembles into the bulk of the archaellum filament.^4,17,18^*M. villosus* is no exception, with 3 archaellins called ArlB1, 2 and 3 encoded in the operon. With this in mind, we modelled the archaellin ArlB2 into our map, which appeared to fit the protomer densities best (Fig. 2e and f). However, the outer regions of the filament were less well resolved, making the backbone harder to trace than in the core.^14^

**Fig. 3:**
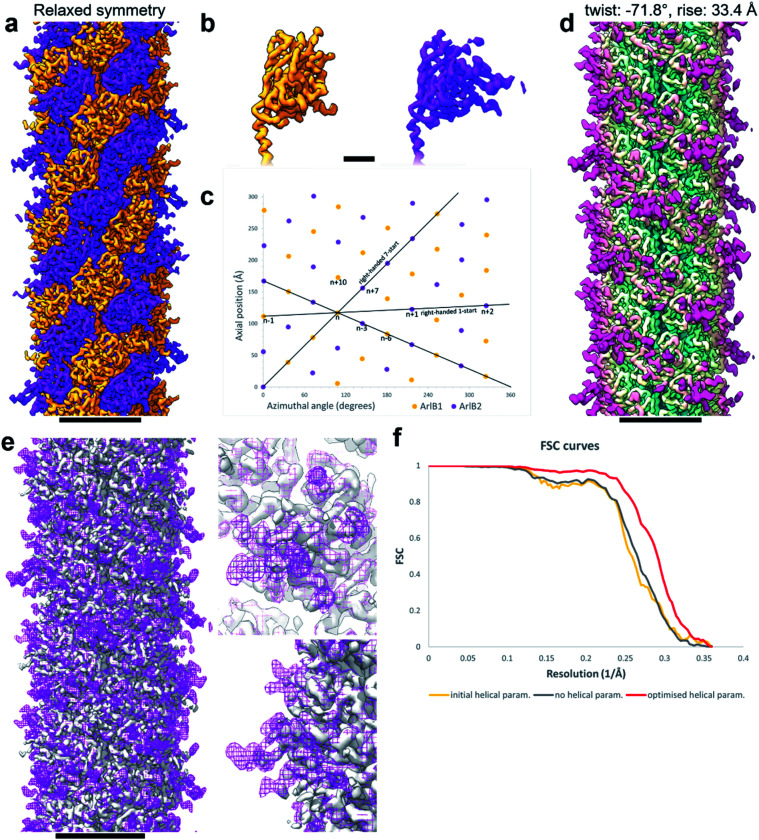
Refining helical parameters after symmetry relaxation. (a) CryoEM map of the *M. villosus* archaellum filament solved with relaxed helical symmetry showing ArlB1 in orange and ArlB2 in purple. (b) Close-up of the head domains of ArlB1 (orange) and ArlB2 (purple). (c) Helical net diagram showing the positions of ArlB1 (orange dots) and ArlB2 (purple dots) in a two-dimensional plot. Solid black lines show various component helices. (d) CryoEM map of the archaellum filament solved imposing helical symmetry with refined parameters of −71.8° twist and 33.4 Å rise. The map is coloured in petrol blue–light yellow–magenta from the core to the periphery of the filament. (e) Superposition of the cryoEM map obtained relaxing the helical symmetry (solid grey) and cryoEM map obtained applying refined helical parameters (mesh magenta). Two close-ups show the peripheral areas best resolved using the refined helical parameters. (f) Fourier shell correlation curves comparison between the cryoEM maps obtained with initial helical parameters (108° twist and 5.5 Å rise; orange), relaxed helical symmetry (grey), refined helical parameters (−71.8° twist and 33.4 Å rise; red). Scale bar in (a, d, e), 50 Å; in (b), 10 Å.

Curious as to whether we could resolve different conformations of the inherently flexible filament, we imported the refined particles into CryoSPARC 3.1.0 to perform 3D variability analysis (3DVA). As the first step of this analysis, a new refinement was performed using the helical refinement (BETA) algorithm without imposing helical parameters. Strikingly, this resulted in a reconstruction with 3.28 Å resolution, where the filament appeared to consist not of one, but two distinct subunits that alternated throughout the filament (Fig. 2g–i, Fig. 3a and b). This was confirmed by a round of 3D refinement with no helical symmetry in Relion 3.1.^14^

Through careful Real Space analysis using a net diagram (Fig. 3c), we found that the originally imposed helical parameters of 5.57 Å rise and 108° twist did not describe the filament’s alternating architecture correctly. Curiously, the filament could not be described as one having *n* + 2 helical symmetry by doubling of its helical parameters to 11.14 Å rise and 216° (−144°) twist. Instead, the minimal transformation of the *M. villosus* archaellum in which each monomer superimposes onto its equivalent was *n* + 6, resulting in revised helical parameters of 33.4 Å rise and −71.8° twist.^13^ Employing these helical parameters in a final round of refinement resulted in a map with improved resolution of 3.08 Å (Fig. 3d and e). The map showed a significantly improved quality of the outer regions compared to the previous one reconstructed with the erroneous helical parameters (Fig. 2e and f) and also compared to the map obtained by helical relaxation (Fig. 3e and f). The densities for glycan moieties that decorate the archaellum were better defined (asterisks in Fig. 2j) and we could unambiguously model the sequences of ArlB1 and ArlB2 into the new map, resulting in the structures shown in Fig. 2j and k.^14^

In the complicated symmetry of the structure, ArlB1 and ArlB2 alternate along the three left-handed 3-start helices that make up the archaellum. Interestingly, every third 3-start helix is out of register with respect to the previous two. The biological significance of this register shift is so far not clear. However, it can be assumed that this shift causes a screw axis asymmetry that may bias the superstructure of the filament into the stable superhelix that is observed in gyrating archaella.^14^

## How to escape the symmetry trap

**Fig. 4:**
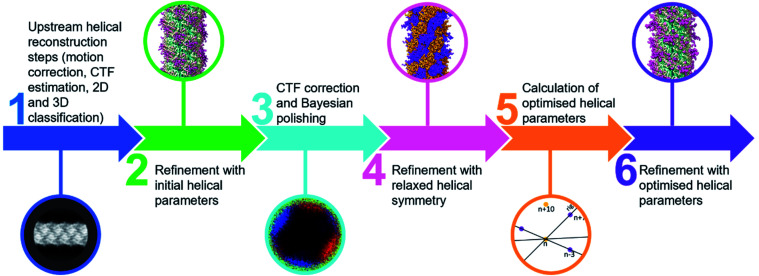
Flow chart for helical processing including a symmetry relaxation step.

Comparing the structures obtained with the incorrect (Fig. 2a–f) and the correct set of helical parameters (Fig. 2g–k, Fig. 3e and f) showcases how complex helical pseudosymmetries can hide within simpler solutions that seemingly make biological sense. To avoid overlooking higher order symmetries in future, we suggest that a symmetry relaxation step should be a default precaution in any helical reconstruction project and propose the workflow shown in Fig. 4. Briefly, symmetry relaxation should occur as a final helical refinement step, in which no helical parameters are imposed to the structure.

As exemplified by our data, it is advisable to perform symmetry relaxation even in cases where the structure appears to be biologically meaningful and shows the structural details that are expected at the resolution. This strategy will potentially uncover unexpected heterogeneities that are averaged out by the imposed symmetry and in addition allow the investigation of filament flexibility, as implemented in CryoSPARC’s 3DVA.^12^ Furthermore, we suggest that the maps resulting from unsymmetrised 3D refinements should be included in the validation process. This could entail a Fourier shell correlation between the symmetrised and unsymmetrised maps (Fig. 3f), as well including the unsymmetrised maps in data deposition to the Unified Data Resource for 3-dimensional Electron Microscopy (EMD).

## Conclusions

Helical reconstruction is inherently dependent on applying high orders of symmetry. As exemplified here, applying seemingly correct symmetry parameters can average out heterogeneities or more complex symmetries. It would therefore be recommendable to include a default symmetry relaxation step during the reconstruction procedure and incorporate the result in the data validation process.

## Conflicts of interest

There are no conflicts of interest to declare.
